# Identification and characterization of agonist epitopes of the MUC1-C oncoprotein

**DOI:** 10.1186/2051-1426-1-S1-P218

**Published:** 2013-11-07

**Authors:** Caroline Jochems, Jo A Tucker, Matteo Vergati, Benjamin Boyerinas, James L Gulley, Jeffrey Schlom, Kwong-Yok Tsang

**Affiliations:** 1 Laboratory of Tumor Immunology and Biology, NCI, Bethesda, MD, USA; 2 Medical Oncology Branch, NCI, Bethesda, MD, USA

## Purpose

The MUC1 tumor-associated antigen is overexpressed in the majority of human carcinomas and several hematologic malignancies. Much attention has been paid to the hypoglycosylated VNTR region of the N-terminus of MUC1 as a vaccine target, and recombinant viral vector vaccines are also being evaluated that express the entire MUC1 transgene. While previous studies have described MUC1 as a tumor-associated tissue differentiation antigen, numerous studies have now determined that the C-terminus of MUC1 (MUC1-C) is an oncoprotein, and its expression is an indication of poor prognosis in numerous tumor types.

## Experimental design

We report here the identification of seven potential CD8+ cytotoxic T lymphocyte epitopes of MUC1: five in the C-terminus and two in the VNTR region, and have identified enhancer agonist peptides for each of these epitopes. These epitopes span HLA-A2 and A3 MHC class I alleles, which encompass two thirds of the population.

## Results

The agonist peptides, compared to the native peptides, more efficiently (a) generate T-cell lines from the peripheral blood mononuclear cells of cancer patients, (b) enhance the production of IFN-γ by peptide-activated human T cells, and (c) lyse human tumor cell targets in an MHC-restricted manner.

## Conclusions

The agonist epitopes described here can be incorporated in various vaccine platforms and for ex vivo generation of human T cells. These studies thus provide the rationale for the T-cell-mediated targeting of the oncogenic C-terminus of MUC1, which has been shown to be an important factor in both drug resistance and poor prognosis for numerous tumor types.

